# Polymorphisms in the IFN*γ*, IL-10, and TGF*β* Genes May Be Associated with HIV-1 Infection

**DOI:** 10.1155/2015/248571

**Published:** 2015-02-24

**Authors:** Felipe Bonfim Freitas, Sandra Souza Lima, Rosimar Neris Martins Feitosa, Vânia Nakauth Azevedo, Marluísa de O. Guimarães Ishak, Ricardo Ishak, Antonio Carlos R. Vallinoto

**Affiliations:** Laboratory of Virology, Biological Sciences Institute, Federal University of Pará, 66075-110 Belém, PA, Brazil

## Abstract

*Objective.* This study investigated possible associations between the TNF*α*-308G/A, IFN+874A/T, IL-6-174C/G, IL-10-1082A/G, and TGF*β*-509C/T polymorphisms with HIV-1 infection, in addition to correlation of the polymorphisms with clinical markers of AIDS progression, such as levels of CD4+/CD8+ T lymphocytes and plasma viral load.* Methods.* A total of 216 individuals who were infected with HIV-1 and on antiretroviral therapy (ART) and 294 individuals from the uninfected control group were analyzed.* Results.* All individuals evaluated were negative for total anti-HBc, anti-HCV, anti-*T. pallidum*, and anti-HTLV-1/2. The polymorphisms were identified by PCR-RFLP. Individuals presenting the IFN+874A allele as well as the AA genotype were more frequent in the HIV-1 infected group compared to the control group (*P* < 0.05), in addition to having lower levels of CD4+ T lymphocytes. The CD8+ T lymphocytes count was significantly lower in individuals with the IL-10-1082 GG genotype. The TGF*β*-509TT genotype was associated with higher plasma viral load.* Conclusions.* The results suggest that the presence of the IFN+874A allele confers susceptibility to HIV-1 infection and a decrease in the number of CD4+ T lymphocytes. In addition, the genotype associated with high serum levels of TGF*β* may be associated with an increase in plasma viral load.

## 1. Introduction

HIV-1 infection leads to a progressive decline in the number of peripheral CD4+ T lymphocytes, dysfunctions in thymic T cells, and changes in the number and function of antigen-presenting cells, such as dendritic cells and monocytes [1, 2].

Although most (70–80%) individuals infected with HIV develop AIDS after years of clinical latency, there is a large amount of variation in the process of pathogenesis in different patients, with or without therapeutic intervention [3]. This interindividual variability in susceptibility to the virus, progression from infection to AIDS, success of transmission, or even in response to antiviral therapy has been attributed to the genetic makeup of the virus, environmental factors, and the existence of genetic variability in multiple host genes [4]. Among these host genes are genes encoding cytokines [5–7]. These cytokines can be functionally classified into proinflammatory (TNF*α*, IL-1, IL-6, IFN*γ*, and IL-8) and anti-inflammatory (IL-4, IL-10, IL-13, IL-1ra, and TGF*β*) types and are important mediators of the immune response that function by binding to specific receptors in response to various stimuli [8]. In the context of HIV-1, genetic cohort studies have shown associations of several single nucleotide polymorphisms (SNPs) with different rates of progression and variation in susceptibility to infection [9], which involves genes encoding molecules that participate in the adsorption process and viral penetration into the host cell (CCR5, CCR2, RANTES, and SDF1), in the regulation of the immune response (IL-10, TNF*α*, and MBL), and in the recognition of epitopes of viral antigens by CD4+ and CD8+ T lymphocytes (human leukocyte antigen or HLA).

Immunogenic profiles have been widely described as important factor associated with susceptibility and evolution of HIV-1 infection, especially those single nucleotide polymorphisms (SNPs) in cytokines genes that regulate the imune response like TNF*α*, IFN*γ*, TGF*β*, IL-10, and IL-6, but few reports have focused patients from the Amazon region of Brazil, where the people have a genetic background composed by the mix of three ethnic groups. Therefore, the present study aimed to evaluate the occurrence of a possible association of TNF*α*-308G/A, IFN*γ*+874T/A, TGF*β*-509C/T, IL-10-1082A/G, and IL-6-174G/C polymorphisms with HIV-1 infection in Brazilians residing in the northern part of country and its influence on laboratory markers of AIDS progression (CD4+ T lymphocytes and plasma viral load).

## 2. Materials and Methods

### 2.1. Examined Populations

A total of 216 investigated subjects who were infected with HIV-1 (130 men and 86 women), with a mean age of 39 ± 10.98 years, and who underwent follow-up at the Reference Unit Specializing in Infectious and Parasitic Diseases (Unidade de Referência Especializada em Doenças Infecciosas e Parasitárias (URE-DIPE)) located in Belém, the capital of the state of Pará, Brazil, were assessed from 2008 to 2010. All seropositive patients were part of the National Network of CD4+ and CD8+ T Cell Count and Plasma Viral Load, which is managed by the Brazilian Ministry of Health, and were undergoing antiretroviral therapy (ART). The control group was comprised of 294 healthy HIV-1 seronegative individuals (191 men and 103 women) with a mean age of 29 ± 9.39 years. The individuals from both patient and control groups had similar ethnic origins and risk for HIV-1 infection and were residents in the same geographical area. In both populations, all subjects who were heterozygous for the CCR5Δ32 [10] deletion were excluded to avoid bias toward HIV-resistance conferred by this mutation. Additionally, in the group of patients, exclusion was done to eliminate the possibility that this genetic condition influences the values of the CD4+/CD8+ T lymphocyte counts and plasma viral loads, making the sample more homogeneous. These data were collected from previous studies in the same patients. This study was approved by the research ethics committee of João de Barros Barreto University Hospital, and all individuals who agreed to participate in the study signed an informed consent form.

### 2.2. Serology, Viral Load, and CD4+ and CD8+ T lymphocyte Counts

All individuals involved in the study were tested for the presence of total anti-HBc (BioMérieux, Lyon, France), anti-HCV, anti-T. pallidum, and anti-HTLV-1/2 (Ortho Clinical Diagnostics, New Jersey, USA) antibodies, and those who were positive for any of the markers were excluded. The viral load was quantified by branched DNA assay (bDNA) (Bayer Corporation, Massachusetts, USA), which has a minimum detection limit of 50 RNA copies/mL. CD4+ and CD8+ T lymphocyte counts were performed by flow cytometry (FacsCount, Becton and Dickinson, USA) using a FACSCountTM Reagents immunomonitoring kit according to the standard protocol recommended by the manufacturer.

### 2.3. Identification of Polymorphisms

DNA was extracted from mononuclear cells from whole peripheral blood using the Gentra Systems Puregene DNA isolation kit (Invitrogen, Minneapolis, USA). Amplification of fragments of the TNF*α*, IFN*γ*, TGF*β*, IL-6, and IL-10 genes was performed by polymerase chain reaction (PCR) followed by identification of polymorphisms using the restriction fragment length polymorphism analysis (RFLP) (Table 1). Subsequently, the amplified products were subjected to electrophoresis in 4% agarose gels containing 5 *μ*L of SYBR Safe DNA gel stain (10 mg/mL) (Invitrogen, Minneapolis, USA).

### 2.4. Statistical Analysis

The distributions of allele and genotype frequencies for each polymorphism in the infected and control groups were estimated by direct counting and compared using the chi-square test or Fisher's exact test in cases where the sample size for a given genotype was lower than 5. Hardy-Weinberg equilibrium was tested using the chi-square test. The associations between genotypes and markers of progression (CD4+, CD8+ T lymphocytes and viral load) were evaluated using the Mann-Whitney test. A 5% significance level (*P* ≤ 0.05) was adopted in all statistical analyses using the software BioEstat 5.0v [11].

## 3. Results

### 3.1. Allelic and Genotypic Frequencies

An analysis of deviations between the observed and expected allele and genotype proportions in both groups revealed that both populations were in Hardy-Weinberg equilibrium (*P* > 0.05) for all polymorphisms studied.

In Table 2, it is shown that the allelic and genotypic distributions of the TNF*α*, IL-10, IL-6, and TGF*β* genes did not differ significantly between the two population groups. However, for the IFN*γ* gene, allele A, which is associated with low serum levels of IFN*γ*, and the homozygous AA genotype were associated with HIV-1 infection (*P* = 0.0172 and *P* = 0.0336, resp.). The odds ratio (OR) showed that individuals carrying the AA genotype had a 1.5 times greater chance of being infected with HIV-1 (*P* = 0.0198), and the presence of the A allele was associated with a 1.3 times greater risk (*P* = 0.0210).

### 3.2. Association of Genotypes with Levels of CD4+ and CD8+ T lymphocytes

**Figure 1:**
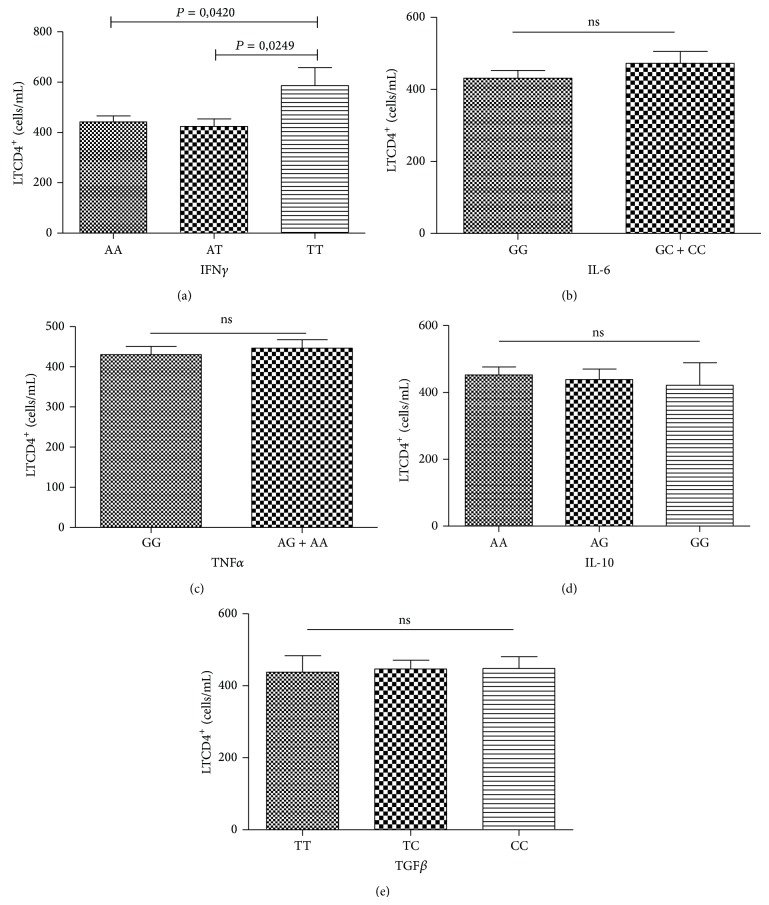
Distribution of CD4+ T lymphocyte counts (cells/mL) by the genotypes of the IFN*γ* (a), IL-6 (b), TNF*α* (c), IL-10 (d), and TGF*β* (e) genes in individuals infected with HIV-1. ns: nonsignificant.

**Figure 2:**
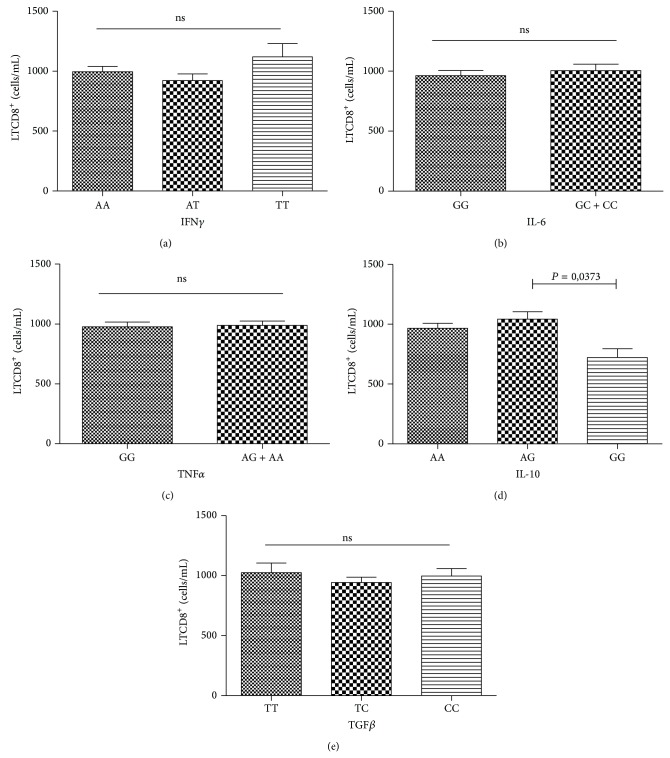
Distribution of CD8+ T lymphocyte counts (cells/mL) by the genotypes of the IFN*γ* (a), IL-6 (b), TNF*α* (c), IL-10 (d), and TGF*β* (e) genes in individuals infected with HIV-1. ns: nonsignificant.

For this analysis, the mean values of the last three CD4+ and CD8+ T lymphocyte counts from each individual were used, which were accessed in the Control System for Laboratory Tests of the National Network of CD4+/CD8+ T Lymphocyte Count and Viral Load (Sistema de Controle de Exames Laboratoriais da Rede Nacional de Contagem de Linfócitos TCD4+/CD8+ e Carga Viral (SISCEL)). The mean count values did not differ significantly between the genotypes of the IL-6, TNF*α*, IL-10, and TGF*β* genes (Figures 1 and 2). However, carriers of the IFN*γ* TT genotype (Figure 1(a)) had significantly higher counts of CD4+ T lymphocytes (586 cells/mL) when compared to carriers of genotypes AA (473 cells/mL) and AT (424 cells/mL).

Individuals carrying the IFN*γ* TT genotype showed higher levels of CD8+ T lymphocytes (1,118 cells/mL) when compared to genotypes AA and AT (983 and 926 cells/mL, resp.), but without statistical significance. The individuals carrying the IL-10 AG (1,032 cells/mL) and AA (955 cells/mL) genotypes had significantly higher levels of CD8+ T lymphocytes compared to those with the IL-10 GG genotype (744 cells/mL) (Figure 2(d)).

### 3.3. Genotypes and Plasma Viral Load

**Figure 3:**
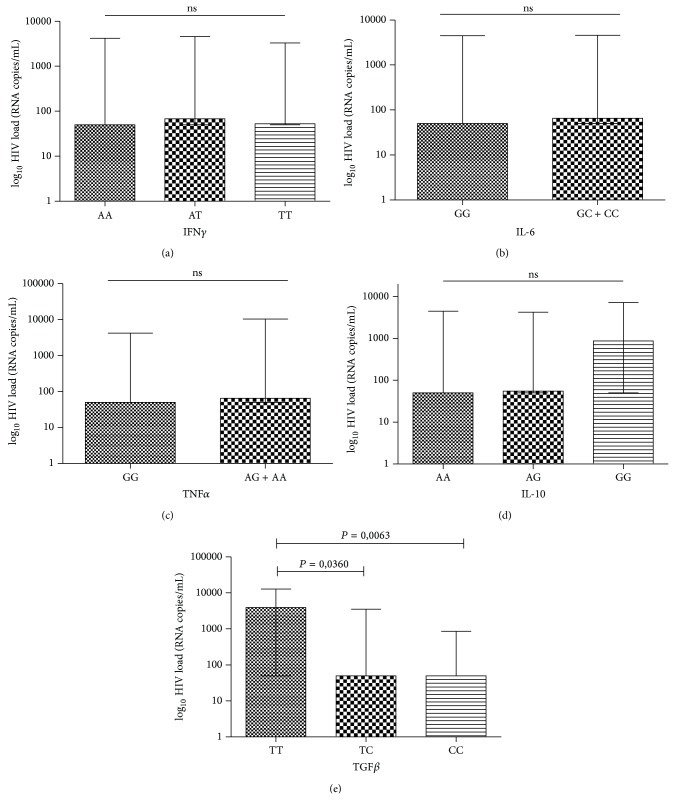
Distribution of HIV-1 plasma viral loads (log⁡_10_⁡) by the genotypes of the IFN*γ* (a), IL-6 (b), TNF*α* (c), IL-10 (d), and TGF*β* (e) genes. ns: nonsignificant.

Following the same pattern of analysis used for the levels of T lymphocytes, where mean of last 3 measurements from each individual was used, the viral load for the various genotypes was carried out to investigate whether there were differences between plasma viral load values and genotypes. Differences in the distribution of genotype frequencies by plasma viral load values were significantly different between genotypes of the TGF*β* gene, and the TGF*β* TT genotype was associated with higher viral load values (Figure 3(e)).

## 4. Discussion

The allelic and genotypic distributions of the genes evaluated in this study show variations that are characteristic of distinct ethnic groups, though a study performed by Kaur et al. [12] in northern India found frequencies similar to those observed in the present study for the IFN*γ*+874T, TNF*α*-308A, IL-6-174C, IL-10-1082G, and TGF*β*-509T alleles. However, other studies have found very distinct frequencies of the IL-6-174C allele in Australians [7], the IL-10-1082G allele in Spaniards [13] and Chinese [14], and the TNF*α*-308A allele in Africans [15]. These differences may be a reflection of different population models, criteria, methods, or sample sizes used in the studies. It is important to note that the genetic contribution of the Amazon population analyzed in this study was characterized using a trihybrid model, with 47% Caucasians, 41% indigenous people, and 12% African descendants [16], and differs from profiles analyzed in other studies, which were formed almost exclusively by single ethnic groups.

In North Americans infected by HIV-1 [17], the frequencies of the IL-10-1082G, IL-4-33T, and IL-2-166T alleles differed significantly only in individuals of African and Hispanic descent, suggesting that some polymorphisms may be indicative of susceptibility and progression of infection by the different population groups. Most genetic studies have been conducted in Caucasians [7, 18–21] and South African populations [15, 22–24]. In Brazil, an association of the TNF*α*-308A allele with susceptibility to HIV-1 infection was observed; however, unlike our study, this was done in the Southeast region of the country and analyzed individuals diagnosed with AIDS who had clinical manifestation of retinitis by CMV [25]; another study, also conducted in the same region, investigated polymorphisms in the genes of IL-18 and IFN*γ* in patients with lipodystrophy; however, no influence of the IFN*γ*-874A allele was found [26].

Some studies have indicated strong associations of the IFN*γ*-874A/T polymorphism in patients infected with HIV-1. It has been suggested that individuals with the homozygous IFN*γ*-874A/A genotype have a significantly higher risk of infection and progression to AIDS [27], similar to the results found here. Likewise in Korean individuals, a significantly higher frequency of the T allele, which is associated with the production of high levels of IFN*γ*, was found in the control group compared to the infected group [28]. Cases of multiple sclerosis in HIV-infected individuals were also associated with the AA genotype [29], suggesting that this genotype may influence the clinical deterioration of these infected individuals.

IFN*γ* is a cytokine produced by T and activated NK cells in response to pathogen invasion. The biological actions of this cytokine are diverse and include immunomodulatory effects, activation of macrophages, and antimicrobial and antiproliferative activities [30]. Binding of IFN*γ* to its receptor activates STAT-1, which in turn binds to transcription factors that will stimulate MHC-II, protein kinase (PKR), and ribonuclease L expression. Thus, blocking or failure of the IFN*γ* signaling pathway confers higher susceptibility to infections, suggesting that secretion of IFN*γ* may be an important effector function for the suppression of HIV-1 and other viral infections [31–33]. In this study, the IFN*γ*+874TT genotype, which is associated with high IFN*γ* production, was related to considerably higher levels of CD4+ T lymphocytes compared to the other two genotypes (AA and AT). Th1 lymphocytes secrete IL-2, IFN*γ*, and TNF*α*/*β* to direct an immune response against intracellular pathogens and stimulate a predominantly cellular immune profile [34]. Additionally, IFN*γ* plays an important role in positive regulation of genes that encode MHC-I, subsequently increasing the potential for cytotoxic T lymphocytes to recognize foreign peptides and thereby promote the induction of cellular immunity [35, 36]. Therefore, high levels of IFN*γ* stimulate the clonal expansion of CD4+ T lymphocytes to promote the maintenance of a response during the course of HIV-1 infection.

The effects and mechanisms involving IL-10 action in the pathogenesis of HIV-1 are quite controversial, suggesting that factors associated with the host, virus, and study models used may lead to these results. Several studies show that alleles associated with low serum levels of IL-10 are linked to increased susceptibility to HIV-1 infection and accelerated progression to AIDS, particularly in later stages of the disease [6, 37, 38]. In contrast, other studies demonstrate a protective role of the alleles that are associated with low levels of IL-10 (-1082A and -592A) [17, 23]. Naicker et al. demonstrated that these alleles conferred susceptibility to HIV infection. In acute infection, low levels could promote protective effects since high levels may promote viral replication by suppressing innate and adaptative immune responses, but in chronic infection stage, high levels of IL-10 may be protective by reducing immune activation and inhibiting HIV replication in macrophages [23]. In animal models, removal of the IL-10 gene or blocking of the activation pathway increases T cell responses, resulting in rapid elimination of the virus and in the development of a response by memory T cells, culminating in virus shedding [39, 40]. The present study found that genotypes associated with low levels of IL-10 (-1082AA and AG) were associated with higher CD8+ T lymphocyte counts compared to genotype -1082GG. Similar to our results, Naicker et al. [22] found that genotype -592AA (low levels) was associated with a greater magnitude of response from virus-specific CD8+ T lymphocytes, suggesting that allelic variants can influence the rate of disease progression by regulating serum levels and modulating the immune response by cytotoxic lymphocytes.

In this study, the TGF*β*-509TT genotype, which is related to high production levels of TGF*β*, was more frequent among individuals with higher plasma viral load levels, even though all patients were undergoing an ART regimen. It is known that TGF*β* stimulates the expression of CTLA-4, inhibiting IL-2 production [41]. The frequency of Treg cells that are positive for CTLA-4 is enhanced in patients with chronic infection with HIV-1, leading to an immune dysfunction associated with the virus [42]. The increased expression of CTLA-4 correlates to disease progression markers, and the in vitro blocking of CTLA-4 increases HIV-specific CD4+ T lymphocytes cellular functions [43]. In addition, TGF*β* may increase the expression of CCR5 and CXCR4, thereby increasing the susceptibility of CD4+ T cells to HIV-1 infection [44].

Thus, our results might suggest that the identification of genetic markers may be required for clinical use and for the stratification of patients into new immunomodulatory treatments. Our results suggest that the predominance of a Th1 over Th2 type response would be ideal to maintain the immune response during HIV-1 pathogenesis and would also influence individual susceptibility to this infection. Our studies suggest that genotypes associated with high levels of IFN*γ* may contribute to the maintenance of a response by CD4+ T lymphocytes in subjects ART and that the TGF*β* suppressor role in the development and maintenance of the immune response would facilitate viral replication resulting in increased levels of viral load. Thus, future studies analyzing other polymorphisms, gene expression, and serum levels will facilitate mapping and identification of candidate genes for diseases. Moreover, one of the biggest challenges that obstruct the development of vaccines is interindividual variability in the immune response to HIV-1.

In Brazil, little is known about the genetic profiles of cytokines in patients infected with HIV-1 or their influence on disease progression. Therefore, this study contributes to evaluate the frequency of these polymorphisms and their influence on biomarkers of progression of HIV infection in this population. However, additional studies are needed to complement the results presented here to better understand all the mechanisms involved in the progression of infection to AIDS.

## Figures and Tables

**Table 1:** Restriction enzymes, primer sequences, annealing temperatures, and fragments generated during polymorphism identification.

Polymorphisms (endonuclease)	Primers	Genome location	Hybridization temperature (°C)	Alleles and fragments (bp)
rs1800795 (Nla III)	IL-6 F: 5′-AAAGGAAGAGTGGTTCTGCTTCT-3′ IL-6 R: 5′-ATCTTTGTTGGAGGGTGAGG-3′	-174G/C (promoter)	59	C: 85, 84 G: 169

rs1800896 (Mnl I)	IL-10 F: 5′-TCTGAAGAAGTCCTGATGTC-3′ IL-10 R: 5′-CTCTTACCTATCCCTACTTCC-3′	-1082A/G (promoter)	60	A: 125, 65 G: 93, 65, 32

rs2430561 (Hinf I)	INFg F: 5′-GATTTTATTCTTACAACACAAAATCAAGAC-3′ INFg R: 5′-GCAAAGCCACCCCACTATAA-3′	+874T/A (first intron)	53	A: 176 T: 148, 28

rs1800629 (Nco I)	TNFa F: 5′-AGGCAATAGGTTTTGAGGGCCAT-3′ TNFa R: 5′-TCCTCCCTGCTCCGATTCCG-3′	-308G/A (promoter)	60	A: 107 G: 87, 20

rs1800469 (Dde I)	TGF*β*1 F: 5′-GGAGAGCAATTCTTACAGGTG-3′ TGF*β*1 R: 5′-TAGGAGAAGGAGGGTCTGTC-3′	-509C/T (promoter)	61	T: 120 C: 74, 46

F: forward; R: reverse; bp: base pairs.

**Table 2:** Distribution of allelic and genotypic frequencies in the genes of IFN*γ*, TNF*α*, IL-10, IL-6, and TGF*β* cytokines in the groups studied.

Genotypes and alleles	HIV-1 (%)	Control (%)	*P* ^a^	OR	*P* ^b^
	*N* = 216	*N* = 294		(95% CI)	
IFN*γ*					
AA	111 (51.39)	117 (39.79)	0.0336	1.5776	0.0198
AT	89 (41.20)	148 (50.35)		(1.09–2.28)	
TT	16 (7.41)	29 (9.86)			
*A *	311 (71)	382 (65)	0.0172	1.3860	0.0210
*T *	121 (29)	206 (35)		(1.05–1.81)	
TNF*α*					
AA	4 (1.86)	6 (2.04)	0.9887	0.9922	0.9416
AG	42 (19.44)	57 (19.39)		(0.25–3.26)	
GG	170 (78.70)	231 (78.57)			
*A *	50 (12)	69 (12)	0.9372	0.9845	0.9843
*G *	382 (88)	519 (88)		(0.66–1.45)	
IL-10					
AA	123 (56.94)	159 (54.09)	0.7645	1.3261	0.5368
AG	79 (36.58)	111 (37.75)		(0.65–2.67)	
GG	14 (6.48)	24 (8.16)			
*A *	325 (75)	429 (73)	0.4139	1.1257	0.4565
*G *	107 (25)	159 (27)		(0.84–1.49)	
IL-6					
GG	142 (65.74)	184 (62.59)	0.1856	2.7011	0.1241
GC	70 (32.41)	96 (32.65)		(0.87–8.38)	
CC	4 (1.85)	14 (4.76)			
*G *	354 (82)	464 (79)	0.2288	1.2129	0.2621
*C *	78 (18)	124 (21)		(0.88–1.66)	
TGF*β*					
TT	43 (19.91)	59 (20.07)	0.2136	0.7959	0.4439
TC	97 (44.91)	152 (51.70)		(0.48–1.31)	
CC	76 (35.18)	83 (28.23)			
*T *	183 (42)	270 (46)	0.2587	0.8656	0.2864
*C *	249 (58)	318 (54)		(0.67–1.11)	

*P* ^a^: analysis between the two groups (Fisher's exact test or chi-square test). *P* ^b^: odds ratio.
